# Special Issue “Sympathetic Nerves and Cardiovascular Diseases”

**DOI:** 10.3390/ijms25052633

**Published:** 2024-02-23

**Authors:** Yutang Wang, Kate M. Denton

**Affiliations:** 1 Discipline of Life Science, Institute of Innovation, Science and Sustainability, Federation University Australia, Ballarat, VIC 3350, Australia; 2 Department of Physiology, Monash University, Melbourne, VIC 3800, Australia; kate.denton@monash.edu; 3 Cardiovascular Disease Program, Monash Biomedicine Discovery Institute, Monash University, Melbourne, VIC 3800, Australia

Cardiovascular diseases (CVDs) constitute a spectrum of disorders affecting the heart and blood vessels, which include coronary heart disease, cerebrovascular disease, and peripheral artery disease [1]. The major underlying cause of CVD is atherosclerosis, which can be exacerbated by risk factors including hypertension, hypercholesterolemia, and diabetes [2].

Despite significant advancements in our understanding of disease pathogenesis and the availability of numerous therapeutic drugs [3], the burden of CVD continues to increase globally [4]. CVDs remain the leading cause of death worldwide, responsible for 17.9 million deaths each year [1]. This suggests that our understanding of CVD pathogenesis remains limited and incomplete. Indeed, a growing body of evidence indicates the significant involvement of the sympathetic nervous system in CVD pathogenesis [5], a notion that has been largely overlooked until recently.

This Special Issue aims to assemble the latest perspectives and research findings to illuminate the role of altered sympathetic nerve activity (SNA) in CVD pathogenesis. It comprises five review articles and six original research articles, collectively emphasizing the significance of SNA in the pathogenesis of various CVDs, including hypertension, atherosclerosis, heart failure, peripheral artery disease, diabetic wound healing, and chronic pain-associated CVDs.

Review 1, titled “Sympathetic Nerve Activity and Blood Pressure Response to Exercise in Peripheral Artery Disease: From Molecular Mechanisms, Human Studies, to Intervention Strategy Development”, explored the abnormal SNA-mediated blood pressure response during exercise in peripheral artery disease. This study elucidates the molecular mechanisms underlying such abnormal responses and intervention strategies to improve them, thus enhancing tolerance and performance during exercise in patients with peripheral artery disease.

Review 2, titled “Stellate Ganglia and Cardiac Sympathetic Overactivation in Heart Failure”, delved into cardiac sympathetic remodeling in stellate ganglia in heart failure. It elucidates potential underlying mechanisms and emphasizes the therapeutic potential of targeting cardiac sympathetic remodeling against malignant cardiac arrhythmias in heart failure.

Review 3, titled “Sympathetic System in Wound Healing: Multistage Control in Normal and Diabetic Skin”, explored sympathetic regulation and signaling pathways in normal and diabetic wound healing. The review highlights the potential utility of β2-adrenoceptor blockers and nicotinic acetylcholine receptor activators in treating diabetic ulcers with neuropathy.

Review 4, titled “Leptin Increases: Physiological Roles in the Control of Sympathetic Nerve Activity, Energy Balance, and the Hypothalamic–Pituitary–Thyroid Axis”, explored the role of leptin in regulating sympathetic nerve activity and energy balance. It also discussed obesity-induced inflammation and its impact on leptin’s actions during obesity.

Review 5, titled “Chronic Pain-Associated Cardiovascular Disease: The Role of Sympathetic Nerve Activity”, explored the link between sympathetic nervous system dysfunction and chronic pain, highlighting its contribution to CVD in chronic pain settings.

The original research articles delve into various aspects related to SNA and its impact on cardiovascular health.

Original Research 1, titled “Aerobic Exercise Prevents Arterial Stiffness and Attenuates Hyperexcitation of Sympathetic Nerves in Perivascular Adipose Tissue of Mice after Transverse Aortic Constriction”, investigated the effect of exercise on arterial stiffness and the potential role of sympathetic nerves within perivascular adipose tissue using the pressure overload-induced heart failure model in mice. This study found that regular aerobic exercise prevented arterial stiffness during heart failure, and sympathetic innervation and adiponectin within PVAT played a role in this process.

Original Research 2, titled “Asprosin in the Paraventricular Nucleus Induces Sympathetic Activation and Pressor Responses via cAMP-Dependent ROS Production”, investigated the roles and underlying mechanisms of asprosin (an adipokine involved in metabolism) in the paraventricular nucleus in regulating sympathetic outflow and blood pressure. The study found that asprosin in the PVN increased sympathetic outflow, blood pressure, and heart rate via NADPH oxidase activation and subsequent superoxide production.

Original Research 3, titled “Relative Contribution of Blood Pressure and Renal Sympathetic Nerve Activity to Proximal Tubular Sodium Reabsorption via NHE3 Activity”, investigated the effects of blood pressure and renal SNA on renal salt and water balance. This study found that an acute increase in blood pressure and renal SNA inhibited hydrogen exchanger 3 (NHE3) activity, thus leading to salt and water excretion.

Original Research 4, titled “Acute Severe Heart Failure Reduces Heart Rate Variability: An Experimental Study in a Porcine Model”, investigated the effect of experimental acute heart failure on heart rate variability. This study found that acute severe cardiac failure, induced by global myocardial hypoxia, was associated with a significant reduction in heart rate variability.

Original Research 5, titled “The Effect of Renal Denervation on T Cells in Patients with Resistant Hypertension”, investigated the effect of Renal Denervation on T-cell signatures in Resistant Hypertension. This study found that patients with resistant hypertension had higher levels of certain types of T cells in the blood, and the extent of the decrease in blood pressure by renal denervation was associated with T cell frequencies at baseline. Therefore, a detailed analysis of T cells might be useful in selecting patients for renal denervation.

Original Research 6, titled “Moxonidine Increases Uptake of Oxidised Low-Density Lipoprotein in Cultured Vascular Smooth Muscle Cells and Inhibits Atherosclerosis in Apolipoprotein E-Deficient Mice”, investigated the effect of the sympatholytic drug moxonidine on atherosclerosis. This study found that moxonidine inhibited atherosclerosis in apolipoprotein E-deficient mice.

Overall, this Special Issue provides a comprehensive overview of the diverse effects of SNA on CVD, from hypertension and atherosclerosis [6] to peripheral artery disease and heart failure [7]. The insights derived from these reviews and original research studies enhance our understanding of the importance of the sympathetic nervous system in CVD pathogenesis and will facilitate the development of new therapies to treat CVDs.
